# Effectiveness of reverse twin block with lip pads-RME and face mask with RME in the early treatment of class III malocclusion

**DOI:** 10.1186/s40510-019-0266-0

**Published:** 2019-04-08

**Authors:** Rohit A. Minase, Wasundhara A. Bhad, Umal H. Doshi

**Affiliations:** 1 Department Of Orthodontics and Dentofacial Orthopaedics, Government Dental College, Nagpur, Maharashtra India; 2 Department of Orthodontics and Dentofacial Orthopaedics, CSMSS Dental College, 68 Builders Society, near Nandanvan Colony, Aurangabad, Maharashtra India

**Keywords:** Reverse twin block, RME, Face mask

## Abstract

**Background:**

The use of face mask for early treatment of class III malocclusion has proven to be successful, but its compliance and related dental side effects have always been a problem. To overcome this, a new approach has been suggested. The purpose of the present study was to compare the effectiveness of reverse twin block with lip pads and fixed rapid maxillary expansion (RTBLP-RME) appliance and face mask with RME (FM-RME) appliance for early treatment of class III malocclusion.

**Methods:**

The sample consisted of 39 patients with class III malocclusion in the age group of 6–12 years (mean 10.17). They were divided into 3 groups of 13 each: reverse twin block with lip pads-RME (RTBLP-RME), face mask with RME (FM-RME), and control group. Treatment time was 9 months. Lateral cephalograms were taken at the start of treatment (T1) and after 9 months (T2) (both groups).

**Results:**

Both appliances were effective in correction of class III malocclusion with significant (*p* < 0.01) changes in all the cephalometric variables except cranial base angulations as compared to the control group. Intergroup comparison showed nonsignificant but greater sagittal changes with RTBLP-RME as compared to the FM-RME group. For all vertical measurements, the RTBLP-RME group showed nonsignificant increase compared to the FM-RME group. Maxillary incisor proclination was less in the RTBLP-RME group than in the FM-RME group, while mandibular incisor proclination was more in the RTBLP-RME group. Condylar inclination was significantly (*p* < 0.01) different for both treatment groups. With the RTBLP-RME group, posterior inclination of the condyle was seen while the FM-RME group showed more forward positioning as compared to the control group.

**Conclusion:**

Both groups were effective in correcting the malocclusion, but RTBLP-RME appliance had nonsignificant but greater impact on maxillary advancement and more hold on the posterior positioning of the mandible with minimal dental compensation as compared to FM-RME appliance.

## Background

A class III malocclusion is a rare malocclusion with a prevalence of 3.4% in Indians and as high as 14% in the Chinese and Japanese population [1]. This malocclusion is easy to recognize but difficult to treat.

Timing of orthodontic treatment in the management of class III malocclusion has always been controversial. To treat this malocclusion during developing years, many interceptive treatment modalities in the form of fixed appliances [2], removable appliances [3], removable functional appliances [4–10], chin cup [11, 12], protraction headgear [13, 14], and skeletal anchorage systems [15] have been proposed.

Out of these approaches, functional appliances like reverse twin block (RTB), Frankel III appliance, removable mandibular retractor (RMR), and orthopedic appliance like face mask are proven to be useful. In a retrospective study, Seehra et al. [16] and Fareen et al. [17] had observed successful correction of developing class III with face mask and reverse twin block therapy. But the skeletal effects like maxillary protraction were more with face mask, while reverse twin block showed mainly dentoalveolar changes.

One of the possible reasons for encouraging results with face mask is the use of rapid expansion with a face mask to loosen the maxilla [13]. Previous studies with Frankel III appliance have shown a similar effect on maxilla which could be because of use of lip pads in the appliance [4]. Thus, it was thought that if rapid expansion along with lip pads can be added to reverse twin block, it could yield more skeletal changes as opposed to conventional design.

Thus, the purpose of this study was to compare the effectiveness of this modified reverse twin block with face mask and untreated controls.

## Materials and methods

**Fig. 1:**
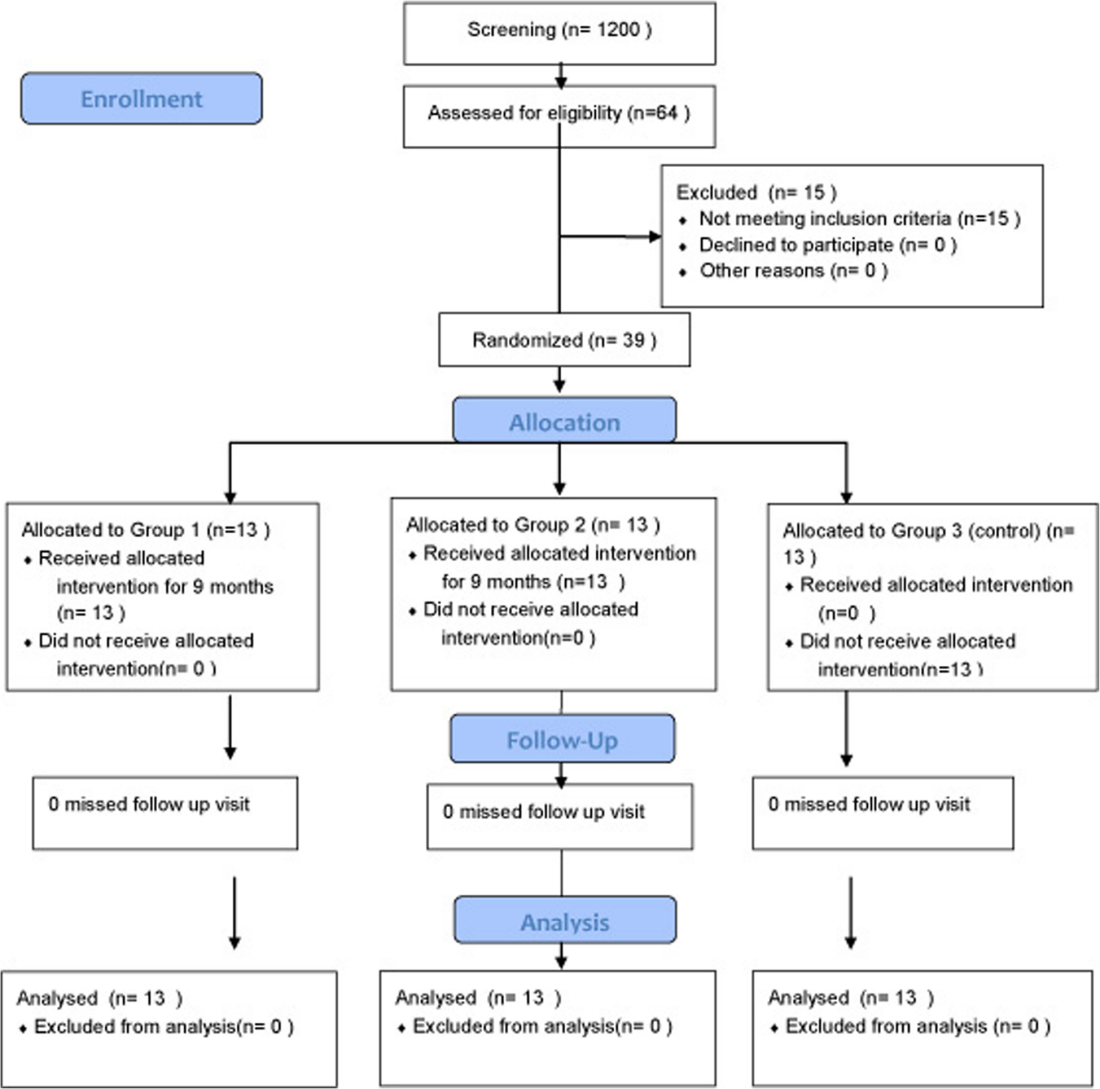
Study flow chart

This was a prospective clinical study with three-arm parallel group randomized clinical trial with a 1:1:1 allocation ratio, conducted on 39 patients with class III malocclusion in the age group of 6–12 years (Fig. 1, Table 1).

**Table 1:** Baseline sample characteristics

	RTBLP-RME group	FM-RME	Control group
Number of patients	13	13	13
Sex distribution	10 females, 3 males	7 females, 6 males	7 females, 6 males
Age (mean ± SD)	10 ± 3.8	10.2 ± 3.7	10.3 ± 3.6
Incisor relation	Anterior crossbite (2 teeth at least)	Anterior crossbite (2 teeth at least)	Anterior crossbite (2 teeth at least)

Before the start of the study, screening ethical approval was sought from the Institutional Ethical Committee under the University of Health Sciences. After ethical approval, screening of 1200 patients reporting at the Government Dental College outpatient department between the age of 6–12 years was done over a period of 3 months. Those children who exhibited class III relationships, assessed primarily on the presence of anterior crossbites (*n* = 64), were further investigated based on following inclusion and exclusion criteria.

### Inclusion criteria

1. Growing patient in the age group 6–12 years
2. Anterior crossbite/edge-edge incisor relationship
3. Angle’s class III molar relation in permanent dentition or mesial step in deciduous dentition
4. Cephalometrically ANB—0° or less (up to − 4°)

### Exclusion criteria

1. Severe skeletal class III resulting primarily from mandibular prognathism (ANB less than − 4°)
2. Class III patients with craniofacial syndromes
3. Cleft lip and palate patients

### Sample size determination

With power of study 80% and 5% significance level, at least 13 samples per group were required. Based on inclusion and exclusion criteria, a total of 39 patients were eligible for the study. These patients were randomly selected and were then assigned to the three groups. Simple randomization was performed by creating a randomization list using Minitab® V16 (Minitab Inc., PA, USA) with an allocation ratio of 1:1:1.

- Group 1: Modified reverse twin block (RTBLP-RME)—13 cases (3 M, 10 F)
- Group 2: Face mask with RME (FM-RME)—13 cases (6 M, 7 F)
- Group 3: Untreated (control)—13 cases (6 M, 7 F)

All patients and/or their parents were apprised of the purpose and procedure of the study, and written consent was taken. For group allocation, gender was not taken into consideration. A control sample was taken for speculation of change due to growth or as a result of therapy.

### Records

In all patients, lateral cephalograms were taken at the start of the treatment (T1) and after 9 months of treatment (T2) in the three groups. In both treatment groups, after 9 months, positive incisor overjet was achieved.

### Appliance design (Fig. 2)

#### Reverse twin block with lip pads-RME

Bite registration was done with maximum mandibular retrusion with 2 mm of inter-incisal clearance and at least 5 mm clearance in the buccal segments to allow sufficient height for the blocks.

**Fig. 2:**
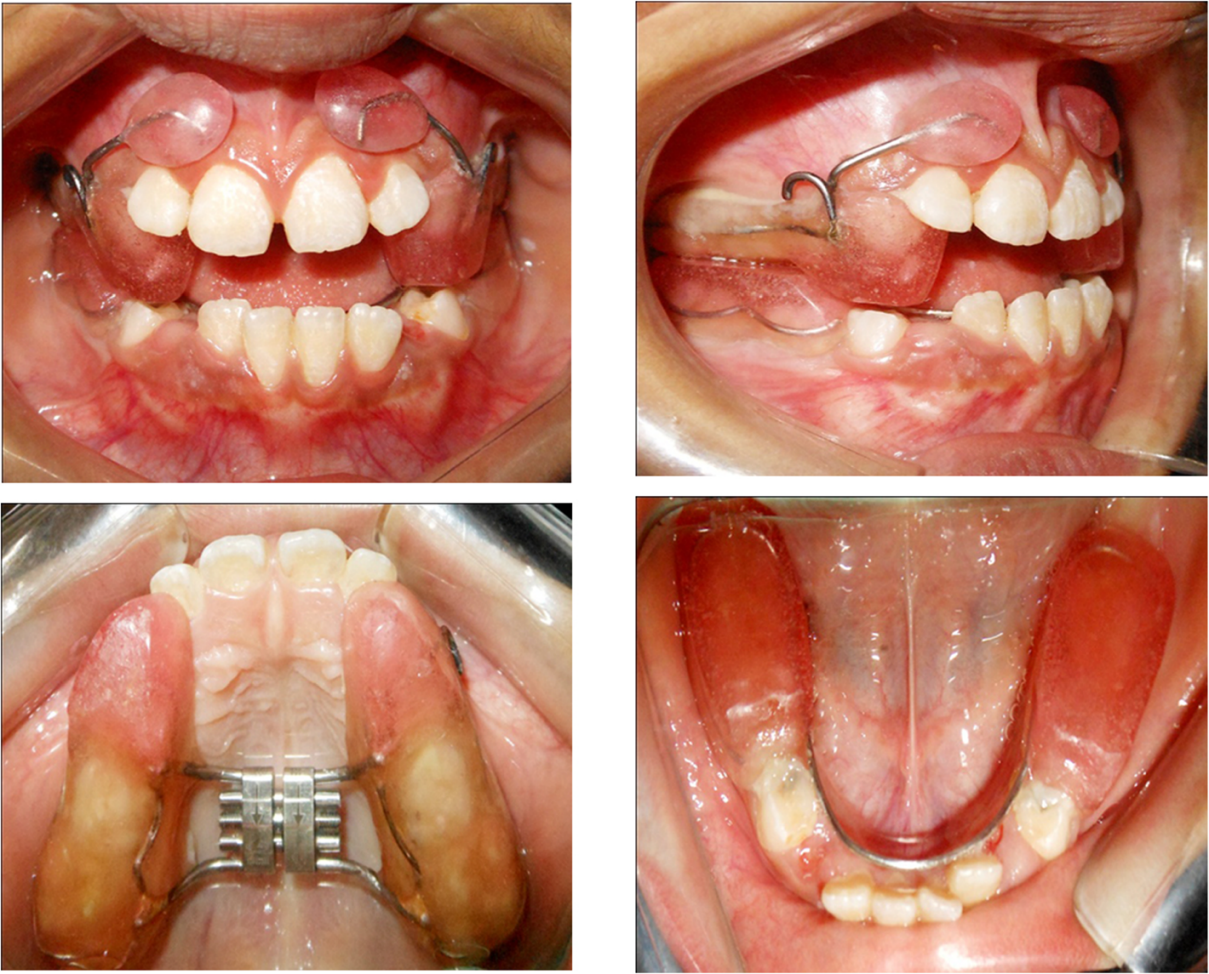
Reverse twin block with lip pads-RME (RTBLP-RME) appliance

Wire framework was made in 0.040-in. stainless steel wire for upper and lower appliance.

HYRAX screw (Leone Orthodontics and Implantology, Firenze, Italy) was adapted parallel to the occlusal plane with arrows directing posteriorly for ease of activation.

Acrylic upper bite blocks were constructed on the occlusal surface extending from canine to first premolar/deciduous canine to deciduous first molar. Lower bite blocks extended from permanent second molar/permanent first molar to second premolar/second deciduous molar. Bite blocks were constructed at 70° to the occlusal plane, configured in reverse of the conventional twin block.

A wax relief of 3-mm thickness was placed between the wire extension for lip pads and plaster model in order to keep it away from the maxilla. They were rhomboidal in shape, 8 mm in height, 12 mm from the incisal edge, and not joined in the midline.

The acrylised, finished, and polished appliance was checked in patient’s mouth and cemented by using glass ionomer type-I luting cement (GC Fuji I).

### Face mask with RME

The design used was as given by Baccetti et al. [18]. A bonded acrylic palatal expansion appliance with HYRAX screw (Leone Orthodontics and Implantology, Firenze, Italy) was constructed with hooks between canine and first premolar/first deciduous molar for attachment of the force delivering elastics. Appliance was cemented using glass ionomer type-I luting cement. A petit face mask was adjusted such that after elastic placement, the direction of force will be 30° downward to the occlusal plane. Initially, for 2 months, a force of 8 oz. per side was given which was increased to 14 oz. per side till the next 7 months. Force was measured with a Dontrix gauge.

### Activation of HYRAX screw

Activation of the HYRAX screw in both treatment groups started from the second day of appliance cementation. On the second day, one full turn was given. Then, from the third day, one turn in the morning and one turn in the evening were given. Activation was done till the slopes of the palatal cusp of maxillary molar coincided with the slopes of the buccal cusp of the mandibular molar.

All patients were reviewed on a 4-week interval basis.

**Fig. 3:**
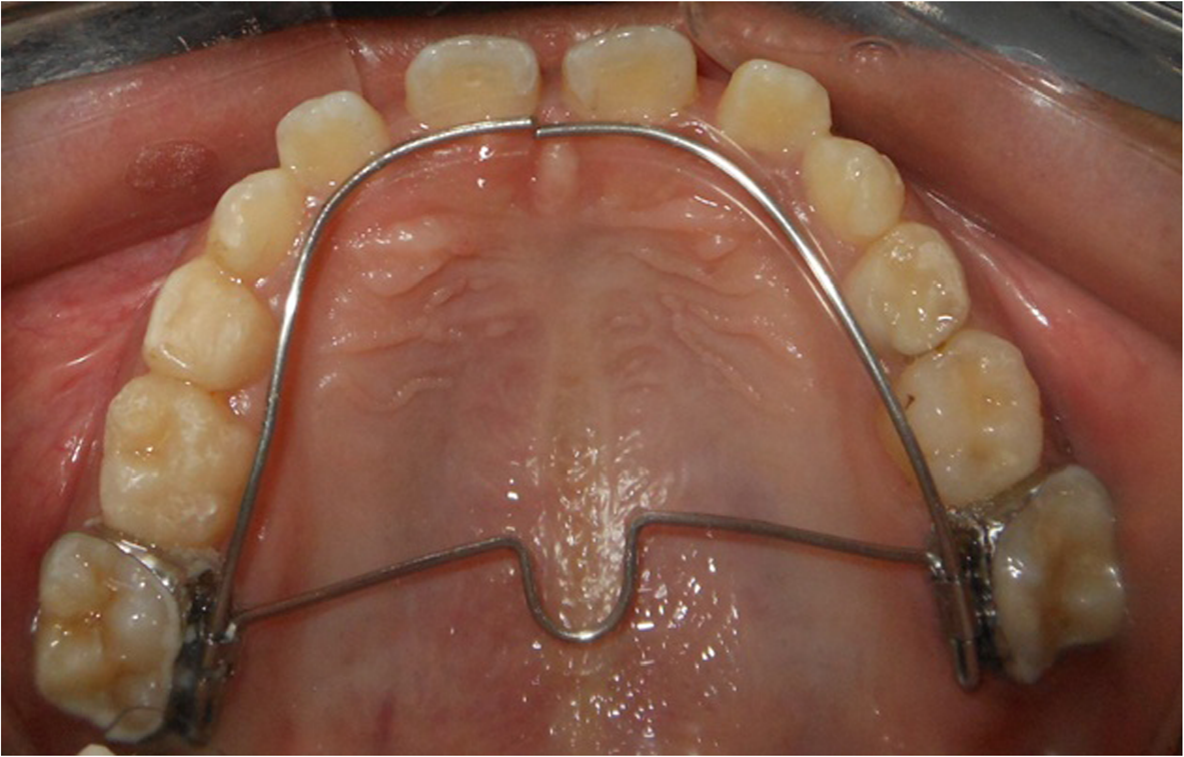
Retention appliance

RTB and PFM were discontinued following the establishment of a positive overjet and overbite, and T2 records were taken at this stage. Immediately after discontinuation of appliances, patients were given fixed modified transpalatal arch (TPA) with anterior extension as retention appliance (Fig. 3).

### Cephalometric analysis

**Fig. 4:**
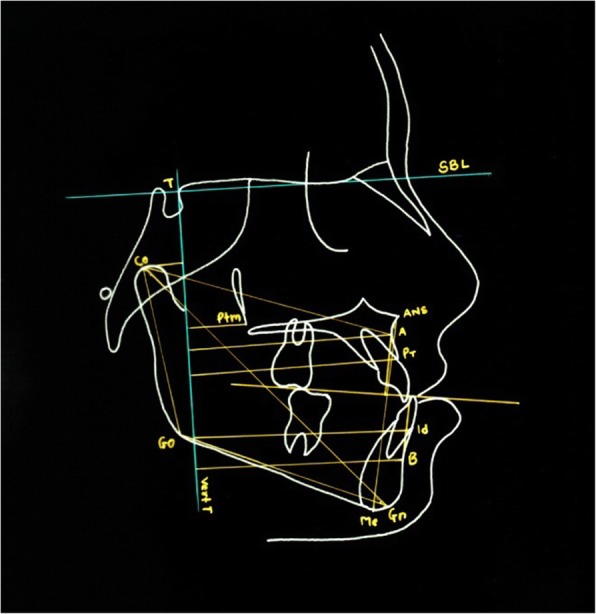
Linear measurements

**Fig. 5:**
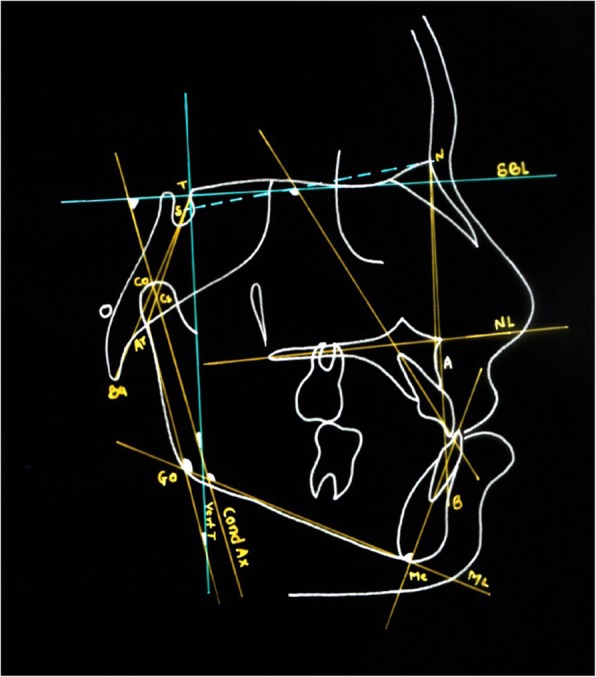
Angular measurements

T1 and T2 cephalograms were taken in the same cephalostat with teeth in habitual occlusion and lips in repose. The enlargement factors (about 5%) were similar among radiographic units; thus, no correction was made for enlargement in the analysis. The cephalometric system described by Baccetti et al. [18] and Tollaro et al. [8] was used. All radiographs were hand-traced on acetate paper for various linear and angular measurements (Figs. 4 and 5).

#### Two reference lines were used

1. Stable basicranial line (SBL)—this line was traced through the most superior point of the anterior wall of the sellaturcica at the junction with tuberculumsella (point T), and it is tangent to lamina cribriosa of the ethmoid.
2. Vertical T (Vert T)—the line perpendicular to SBL and passing through point T.

Using these reference planes, various linear and angular measurements were done (Table 2) (Figs. 3 and 4).

**Table 2:** Definitions of angular and linear measurements used in this study

Measurement	Definition
Ba-T-Vert T	Angle between basion, tuberculumsella, and vertical T line
Ar-T-Vert T	Angle between articulare, tuberculumsella, and vertical T line
SNA	The angle between the anterior cranial base and NA plane
SNB	The angle between the anterior cranial base and NB plane
ANB	SNA minus SNB
WITS	Distance between points A and B projected as perpendicular lines along the functional occlusal plane
A-Vert T	Distance between point A and vertical T line
Ptm-Vert T	Distance between pterygomaxillary fissure and vertical T line
Co-A	Distance between condylion and vertical T line
Pr-Vert T	Distance between pronasale and vertical T line
B-Vert T	Distance between point B and vertical T line
Id-Vert T	Distance between infradentale and vertical T line
Co-Gn	Distance between condylion and gnathion
Co-Go	Distance between condylion and gonion
Go-Gn	Distance between gonion and gnathion
Co-Vert T	Distance between condylion and vertical T line
ML-SBL	Angle between mandibular line and stable basicranial line
NL-SBL	Angle between nasal line and stable basicranial line
NL-ML	Angle between nasal line and mandibular line
Ar-GO-Me	Angle between articulare, gonion, and menton
ANS-Me	Distance between anterior nasal line and menton
UI-SN	Distance between upper incisor and vertical T line
IMPA	Incisor mandibular plane angle
CondAx-Vert T	Angle between condylar axis and vertical T line
CondAx-SBL	Angle between condylar axis and stable basicranial line
CondAX-ML	Angle between condylar axis and mandibular line
Ar-Go-Vert T	Angle between articulare, gonion and vertical T line

### Statistical analysis

Tracings of all lateral cephalograms were done by one observer to avoid inter-examiner bias. Repeat tracings of all cephalograms were done after 2 weeks to determine the method error using Dahlberg’s formula, and it was negligible.

One-way analysis of variance (ANOVA) was used to compare treatment effects between the three groups. Differences between individual groups were assessed by post hoc analysis. All data analysis was performed with the Statistical Package for the Social Sciences (SPSS Inc., version 22.0, IBM Corp, Armonk, NY, USA) with a pre-specified level of statistical significance of *p* < 0.05.

## Results (Tables 3, 4, and 5)

The pretreatment cephalometric parameters in all the three groups, i.e., RTBLP-RME, FM-RME, and control group, showed a non-significant difference (Table 3) suggesting that at pretreatment malocclusion was almost similar.

**Table 3:** Pre-treatment comparison (T1) of the mean values of cephalometric analysis between RME-RTB, RME-FM, and control group

Parameters	RME-RTB (*n* = 13), *X* ± SD	RME-FM (*n* = 13), *X* ± SD	Control (*n* = 13), *X* ± SD		*p* value		
				*p* value	RME-RTB, RME-FM	RME-RTB control	RME-FM control
Measurements for assessment of cranial base angulations							
< Ba-T-Vert T	31.15 ± 3.97	32.23 ± 4.76	31.07 ± 4.46	0.756	0.806	0.993	0.786
< Ar-T-Vert T	26.53 ± 4.09	27.61 ± 2.90	26.07 ± 4.27	0.574	0.753	0.944	0.569
Antero-posterior skeletal measurements							
< SNA	79.15 ± 3.18	78.92 ± 3.14	78.46 ± 2.10	0.824	0.974	0.813	0.918
< SNB	82.23 ± 2.71	81.46 ± 2.84	81.50 ± 2.10	0.697	0.723	0.752	0.998
< ANB	− 3.07 ± 1.65	− 2.53 ± 2.06	− 3.03 ± 1.36	0.675	0.704	0.992	0.746
Wits (mm)	− 8.15 ± 2.37	− 7.23 ± 2.24	− 7.50 ± 2.50	0.596	0.589	0.761	0.952
A-Vert T (mm)	63.76 ± 6.39	60.38 ± 6.14	63.42 ± 4.44	0.261	0.306	0.987	0.372
Ptm-Vert T (mm)	18.07 ± 2.92	15.76 ± 2.77	18.15 ± 2.19	0.042*	0.085	0.992	0.072
Co-A (mm)	83.38 ± 5.82	81.38 ± 7.93	80.69 ± 5.32	0.544	0.714	0.543	0.962
Pr-Vert T (mm)	67.23 ± 6.83	62.38 ± 7.19	65.53 ± 4.29	0.146	0.133	0.775	0.414
B-Vert T (mm)	67.53 ± 8.57	61.07 ± 9.34	66.46 ± 6.27	0.118	0.122	0.946	0.225
Id-Vert T (mm)	69.38 ± 7.98	62.69 ± 9.18	67.69 ± 6.68	0.104	0.097	0.855	0.266
Co-Gn (mm)	114.69 ± 8.71	110.07 ± 10.1	110 ± 6.97	0.302	0.372	0.363	0.962
Co-Go (mm)	53.30 ± 4.19	52.84 ± 7.39	50.84 ± 4.86	0.501	0.975	0.517	0.641
Go-Gn (mm)	76.30 ± 6.70	72.23 ± 7.63	72.61 ± 4.95	0.224	0.264	0.339	0.986
Co-Vert T (mm)	15.46 ± 3.16	16.53 ± 2.87	13.61 ± 2.50	0.047*	0.603	0.242	0.032*
Vertical skeletal measurements							
< ML-SBL	26.92 ± 5.04	26.76 ± 7.22	26.23 ± 6.22	0.955	0.995	0.952	0.977
< NL-SBL	− 0.15 ± 1.99	1.23 ± 4.93	0.76 ± 1.09	0.525	0.504	0.731	0.928
< NL-ML	27.15 ± 4.82	25.53 ± 3.35	25.46 ± 5.48	0.586	0.65	0.626	0.999
< Ar-Go-Me	131.84 ± 4.82	129.69 ± 5.67	133.15 ± 3.67	0.198	0.498	0.768	0.173
ANS-Me (mm)	63.30 ± 5.36	59.84 ± 4.18	59.15 ± 4.33	0.063	0.156	0.079	0.922
Dentoalveolar measurements							
< UI-SN	110.53 ± 6.10	106.84 ± 9.80	105.38 ± 10.4	0.337	0.552	0.321	0.917
< IMPA	85.23 ± 5.67	85.38 ± 9.15	84.53 ± 9.23	0.96	0.99	0.972	0.963
Measurements for assessment of condyle inclination							
< CondAx-Vert T	20 ± 4.47	20.92 ± 8.75	23.46 ± 3.23	0.321	0.91	0.31	0.533
< CondAx-SBL	71.46 ± 5.01	70.53 ± 8.81	66.76 ± 2.48	0.126	0.926	0.136	0.263
< CondAx-ML	136.23 ± 5.05	136.61 ± 7.64	139.23 ± 5.64	0.414	0.985	0.446	0.532
< Ar-Go-Vert T	16.53 ± 4.77	14.38 ± 6.27	19.69 ± 2.78	0.026*	0.494	0.239	0.024*

*Significant

**Table 4:** Post-treatment comparison (T2) of the mean values of cephalometric analysis between the RME-RTB, RME-FM, and control groups

Parameters	RME-RTB (*n* = 13), *X* ± SD	RME-FM (*n* = 13), *X* ± SD	Control (*n* = 13), *X* ± SD	*p* value	*p* value		
					RME-RTB, RME-FM	RME-RTB control	RME-FM control
Measurements for assessment of cranial base angulations							
< Ba-T-Vert T	30.76 ± 2.80	32.38 ± 4.55	31.30 ± 4.19	0.567	0.552	0.936	0.768
< Ar-T-Vert T	26.69 ± 3.06	27.61 ± 3.92	26 ± 3.71	0.524	0.791	0.872	0.499
Antero-posterior skeletal measurements							
< SNA	81.15 ± 2.79	80.23 ± 3.46	79.26 ± 2.04	0.243	0.684	0.217	0.669
< SNB	81.15 ± 3.31	80.73 ± 2.99	82.88 ± 2.27	0.142	0.923	0.299	0.152
< ANB	0.00 ± 1.08	− 0.50 ± 1.93	− 3.61 ± 1.34	0.008**	0.674	0.006**	0.005**
Wits (mm)	− 3.92 ± 3.56	− 4.84 ± 2.19	− 8.53 ± 2.40	0.009**	0.678	0.005**	0.006**
A-Vert T (mm)	66.80 ± 5.92	62.88 ± 5.80	65.15 ± 4.50	0.192	0.179	0.724	0.547
Ptm-Vert T (mm)	18.80 ± 3.05	16.30 ± 2.68	18.76 ± 2.42	0.038*	0.063	0.992	0.063
Co-A (mm)	87.73 ± 4.80	85.84 ± 7.53	82.46 ± 5.75	0.096	0.712	0.085	0.344
Pr-Vert T (mm)	69.76 ± 7.03	65.30 ± 6.48	67.07 ± 4.44	0.182	0.169	0.506	0.748
B-Vert T (mm)	67.15 ± 9.29	61.30 ± 8.41	69.30 ± 6.48	0.048*	0.172	0.783	0.045
Id-Vert T (mm)	69.15 ± 9.29	63.46 ± 7.86	70.76 ± 6.40	0.064	0.177	0.862	0.066
Co-Gn (mm)	119.07 ± 9.16	115.92 ± 10.34	113.61 ± 8.08	0.333	0.662	0.367	0.804
Co-Go (mm)	56.30 ± 4.78	56.76 ± 7.66	52.23 ± 5.70	0.136	0.986	0.225	0.163
Go-Gn (mm)	78 ± 6.63	75.61 ± 8.06	74.15 ± 5.25	0.358	0.647	0.325	0.846
Co-Vert T (mm)	16.73 ± 2.42	17.84 ± 2.79	14.03 ± 2.76	0.008**	0.546	0.035*	0.004**
Vertical skeletal measurements							
< ML-SBL	27.69 ± 5.46	27.53 ± 6.29	25.92 ± 6.38	0.714	0.991	0.734	0.771
< NL-SBL	− 0.69 ± 2.17	1.23 ± 3.58	0.30 ± 1.60	0.185	0.153	0.598	0.634
< NL-ML	28.46 ± 4.42	26.30 ± 4.21	25.61 ± 5.53	0.292	0.488	0.295	0.927
< Ar-Go-Me	132.46 ± 4.46	128.92 ± 5.79	134.69 ± 4.81	0.027*	0.185	0.508	0.016*
ANS-Me (mm)	67.38 ± 6.07	63.30 ± 4.69	60.69 ± 4.40	0.006**	0.114	0.004**	0.408
Dentoalveolar measurements							
< UI-SN	113.61 ± 5.51	112.30 ± 7.83	107.76 ± 10	0.164	0.902	0.168	0.322
< IMPA	85.46 ± 6.33	84.76 ± 8.91	81.92 ± 9.07	0.519	0.971	0.529	0.657
Measurements for assessment of condyle inclination							
< CondAx-Vert T	22.15 ± 3.69	19.30 ± 6.35	24.23 ± 4.08	0.045*	0.304	0.528	0.033*
< CondAx-SBL	69.30 ± 4.97	72.92 ± 7.34	66.92 ± 3.40	0.028*	0.221	0.512	0.022*
< CondAx-ML	139.30 ± 6.87	135.38 ± 8.18	139.69 ± 4.80	0.219	0.319	0.988	0.249
< Ar-Go-Vert T	14.84 ± 5.45	12.61 ± 4.35	20.46 ± 2.43	0.008**	0.383	0.009**	0.003**

*Significant

**Highly significant

**Table 5:** Changes occurring in each group during the study period (T2-T1)

Parameter	RME-RTB, (*n* = 13), *X* ± SD	RME-FM (*n* = 13), *X* ± SD	Control (*n* = 13), *X* ± SD	*p* value	RME-RTB vs RME-FM *p* value
Measurements for assessment of cranial base angulations					
< Ba-T-Vert T	− 0.38 ± 2.02	0.15 ± 1.95	0.23 ± 1.23	0.637	0.723
< Ar-T-Vert T	0.15 ± 2.54	0.00 ± 1.68	− 0.08 ± 0.95	0.942	0.979
Antero-posterior skeletal measurements					
< SNA	2 ± 1.15	1.31 ± 1.05	0.81 ± 0.56	0.018*	0.174
< SNB	−1.08 ± 1.38	− 0.73 ± 1.45	1.38 ± 0.76	0.009**	0.757
< ANB	3.08 ± 1.55	2.04 ± 1.08	− 0.58 ± 0.49	0.003**	0.064
Wits (mm)	4.23 ± 2.03	2.38 ± 1.38	− 1.04 ± 0.72	0.007**	0.005**
A-Vert T (mm)	3.04 ± 1.12	2.50 ± 1.41	1.73 ± 0.88	0.023*	0.477
Ptm-Vert T (mm)	0.73 ± 1.01	0.54 ± 0.77	0.62 ± 0.76	0.845	0.839
Co-A (mm)	4.35 ± 2.51	4.46 ± 0.87	1.77 ± 0.72	0.005**	0.982
Pr-Vert T (mm)	2.54 ± 1.45	2.02 ± 1.70	1.54 ± 0.77	0.037*	0.756
B-Vert T (mm)	− 0.38 ± 3.2	0.23 ± 2.58	2.85 ± 1.51	0.003**	0.819
Id-Vert T (mm)	− 0.23 ± 3.8	0.77 ± 2.68	3.08 ± 1.44	0.15	0.643
Co-Gn (mm)	4.38 ± 2.66	5.85 ± 2.44	3.62 ± 1.32	0.048*	0.226
Co-Go (mm)	3 ± 2.19	3.92 ± 0.95	1.38 ± 1.04	0.005**	0.273
Go-Gn (mm)	1.69 ± 2.21	3.38 ± 3.64	1.54 ± 1.05	0.138	0.212
Co-Vert T (mm)	1.27 ± 1.56	1.31 ± 1.03	0.42 ± 0.53	0.093	0.996
Vertical skeletal measurements					
< ML-SBL	0.77 ± 2.94	0.77 ± 1.73	− 0.31 ± 1.54	0.356	0.967
< NL-SBL	− 0.54 ± 1.85	0.00 ± 2.27	− 0.46 ± 1.26	0.722	0.734
< NL-ML	1.31 ± 2.72	0.77 ± 2.71	0.15 ± 2.07	0.518	0.852
< Ar-Go-Me	0.62 ± 2.50	− 0.77 ± 1.36	1.54 ± 3.82	0.117	0.417
ANS-Me (mm)	4.08 ± 2.32	3.46 ± 3.01	1.54 ± 1.05	0.028*	0.774
Dentoalveolar measurements					
< UI-SN	3.08 ± 7.45	5.46 ± 5.22	2.38 ± 1.71	0.31	0.494
< IMPA	0.23 ± 5.27	− 0.62 ± 4.64	− 2.62 ± 1.50	0.21	0.865
Measurements for assessment of condyle inclination					
< CondAx-Vert T	2.15 ± 4.41	− 1.62 ± 4.68	0.77 ± 2.86	0.077	0.069
< CondAx-SBL	− 2.15 ± 5.06	2.38 ± 4.51	0.15 ± 2.67	0.036*	0.023*
< CondAx-ML	3.08 ± 4.23	− 1.23 ± 3.98	0.46 ± 3.20	0.023*	0.018*
< Ar-Go-Vert T	− 1.69 ± 3.09	− 1.77 ± 2.52	0.77 ± 2.08	0.029*	0.998

*Significant

**Highly significant

### Post-treatment comparison of changes during the study period

#### Cranial Base angulations

The changes in cranial base angulations were non-significant during the study period.

### Antero-posterior skeletal measurements

Significant sagittal improvement (*p* < 0.01) in the form of an increase in SNA, Wits appraisal, and a decrease in SNB was seen with both treatment approaches as compared to the control group. Maximum improvement in SNA was seen with RTBLP-RME group (2°) while FM-RME group showed non-significant improvement (1.31°). Non-significant but greater mean changes in ANB and Wits were seen with RTBLP-RME group (3°, 4 mm) as compared to FM-RME group (2°, 2.4 mm).

Linear measurements showed significant forward repositioning of the maxilla (A-Vert T) (*p* < 0.01) with both appliances as compared to the control group. The RTBLP-RME group showed greater but non-significant sagittal movement (3 mm) as compared to FM-RME group (2.5 mm). Similar changes were seen with Ptm-Vert T and Pr-Vert T measurements with RTBLP-RME group showing slightly more (0.7, 2.05 mm) sagittal forward movement as compared to FM-RME group (0.5, 2.02 mm).

During the treatment period, the changes in B-Vert T were significant with a slight decrease in the RTBLP-RME group (− 0.38) while a slight increase was in the FM-RME (0.23) and control groups (2.85). Similarly, the changes in Co-Vert T were also significant with mean values of 1.27, 1.31, and 0.42 respectively. Mean change Id-Vert T in RTBLP-RME group (− 0.23), FM-RME group, (0.77) and control group (3.08), showing more dentoalveolar remodeling with RTB.

Total mandibular length Co-Gn increased in all the three groups. A highly significant change was seen in ramus length Co-Go with RME-RTB (3.0), RME-FM (3.92), and control group (1.38). However, between the treated groups, no significant change was observed.

Mandibular body length Go-Gn increased with all three groups with the PFM (3.38) group showing more increase as compared to the RTB (1.69) and control groups (1.54).

### Vertical skeletal measurements

In the vertical direction, increase in lower anterior face height was seen with both appliances, while gonial angle (Ar-Go-Me) opened up significantly more (*p* < 0.01) with RTBLP-RME group as compared to FM-RME and Control group.

### Measurements for assessment of condyle inclination

Posterior inclination of condyle was seen with RTBLP-RME group, while FM-RME group showed significantly (*p* < 0.01) more forward positioning as compared to the control group.

During the study period, improvement in antero-posterior direction was slightly more with RTBLP-RME group as compared to FM-RME group. But the difference was statistically non-significant.

### Dentoalveolar measurements

Dental compensation in the form of proclination of maxillary incisors (< UI-SN) was more with FM-RME group (5.4°) as compared to RTBLP-RME group (3.08°). But the difference was non-significant.

Retroclination of lower incisors was seen with FM-RME (IMPA = − 0.62°) while the RTBLP-RME group showed slight proclination (IMPA = 0.23°).

Condylar inclination was significantly (*p* < 0.01) different for both the groups. With the RTBLP-RME group, posterior inclination of condyle was seen while FM-RME group showed more forward positioning as compared to the control group.

## Discussion

Growing class III malocclusions are often treated by extraoral forces to correct the developing skeletal Class III malocclusion. The results are often encouraging, but the problem of patient compliance is a major hurdle in achieving successful results [13, 14]. The other treatment modality for correction of class III malocclusion is reverse twin block (RTB) which is intraoral functional appliance. Studies have reported that RTB effects are primarily dental, characterized by upper incisor proclination and lower incisor retroclination with minimal skeletal change [6, 7, 16, 17].

Conventional RTB was modified in this study by the incorporation of HYRAX screw and upper lip pads with an objective to achieve maximum skeletal effects and minimal dental side effects. A study by Turley PK [19] showed that rapid expansion enhances the protraction effect which in turn reduces the treatment time and produces lesser dental effects. The flaring of the upper incisors is limited because of the space created by the expansion appliance. It also brings about transverse skeletal expansion thus overcoming the transverse maxillary deficiency and also overcomes the resistance of the circummaxillary sutures [19].

Upper lip pads hold the upper lip away, thus eliminating restrictive pressure on the underdeveloped maxilla and also exert tension on the tissues and periosteal attachment at the depth of maxillary sulcus to stimulate bone growth [5].

In present study, RTBLP-RME appliance was cemented to lessen the chance of relapse of expansion. The correction of class III was achieved in a short duration of 9 months in both the treatment groups.

### Effects on cranial base angulation

Over a period of 9 months in the treatment and the control group, no significant changes were observed in cranial base angulations.

### Antero-posterior skeletal measurements

The RTBLP-RME group had a greater impact on maxillary advancement with a mean change in SNA of 2.0 as compared to the FM-RME group (1.31) and control group (0.81).

Changes in angle SNB in RTBLP-RME group (− 1.08), FM-RME group (− 0.73), and control group (1.38) showed that RTBLP-RME has more posterior holding effect/rotational effect on mandibular position than the FM-RME and control groups.

Thus, the mean change in resultant angle ANB for RTBLP-RME group (3.08 ± 1.55) was increased as compared to FM-RME group (2.04 ± 1.08). In the control group, the angle ANB decreased (− 0.58 ± 0.49) indicating that class III worsens with growth.

These effects were in contrast to studies by Kidner et al. [6] and Seehra et al. [7, 16] who had reported minimal effect on SNA, SNB, and ANB. This difference could be attributed to the modification of the reverse twin block done in the present study. Similar positive findings were seen with removable mandibular retractor (RMR) by Saleh et al. [9] and Majanni et al. [10] But in the present study, the amount of sagittal changes seen are more as compared to that in removable mandibular retractor (RMR). This could be attributed to additional components added to reverse twin block in the present study.

Studies on the efficacy of protraction face mask [12–18] have reported similar findings.

Wits appraisal showed greatest mean change with the RTBLP-RME group (4.23) as compared to the FM-RME group (2.83) which was highly significant (< 0.01) again indicating that RTBLP-RME has more positive effect on anteroposterior maxillomandibular relationship in class III malocclusion.

Similar findings have been reported in studies with face mask therapy as compared to control group [13–19]. None of the reverse twin block studies has assessed this parameter.

Mean change in A-Vert T in RTBLP-RME group was (3.04) which was greater than that in the FM-RME group (2.50) and control group (1.73) thus showing that RTBLP-RME appliance is more effective in maxillary advancement. Similar improvement in maxillary advancement has also been noted with removable mandibular retractor (RMR) [8–10] appliance.

Baccetti et al. [18] studied the effect of expansion face mask combination in early and late mixed dentition and found mean change in A-Vert T of 3.58 and 1.89, respectively, thus showing both appliances had nearly the same positive effect on midfacial length as compared to the control group.

Similar findings were recorded by Tortop et al. [20] and Masucci et al. [21] with a mean difference of (2.1) and (8.3), respectively, with face mask treatment.

Mean change of Pr-Vert in RTB (2.54), PFM (2.92), and control group (1.54) indicated that dentoalveolar maxillary protrusion was slightly more with FM-RME group as compared with RTBLP-RME group and least in the control group. A study by Baccetti et al. [17] showed similar findings in early (4.14) and late (2.37) face mask-treated group.

During the treatment period, the changes in B-Vert T were significant for RTBLP-RME group (− 0.38), FM-RME group (0.23), and control group (2.85). Similarly, the changes in Co-Vert T were also significant with mean values of 1.27, 1.31, and 0.42, respectively. These findings support the fact that RTBLP-RME has more hold on the posterior positioning of the mandible.

A study by Baccetti et al. [18] showed a more significant change in B-Vert T in early (− 1.13) and late (− 1.92) treated groups with face mask.

Mean change Id-Vert T in the RTBLP-RME group (− 0.23), FM-RME group (0.77), and control group (3.08) showed more dentoalveolar remodeling with RTBLP-RME. A study by Baccetti et al. [18] showed significant change in early treated (− 0.29) group and late-treated group (− 1.41) with face mask treatment.

Total mandibular length Co-Gn in the overall comparison between the three groups was significant, similar to Baccetti et al. [18], Tortop et al. [20], and Masucci et al. [21]^.^with face mask therapy.

A highly significant change was seen in ramus length Co-Go with mean change in RMELP-RTB group (3.0), FM-RME group (3.92), and control group (1.38). However, within the treated groups, no significant change was observed.

These findings are in contrast to studies by Saleh et al. [9] and Majanni et al. [10] which were with removable mandibular retractor (RMR), and there was no significant difference as compared to control group. Baccetti et al. [18] also found no difference in early versus late treated face mask groups.

Mandibular body length Go-Gn showed mean change with RTBLP-RME group (1.69), FM-RME group (3.38), and control group (1.54). Thus, comparing the effect of RTBLP-RME and FM-RME on mandible, RTBLP-RME showed a significant control on mandibular skeletal base.

### Measurements for vertical relationship

The mandibular plane rotation (ML-SBL) was almost similar in both the treated groups with mean change (0.77°) where as in the control group (− 0.31°), closure of this plane was observed.

Palatal plane inclination (NL-SBL) remained unchanged in FM-RME group (0.00), but in the RTBLP-RME group (− 0.54) it tipped upward anteriorly.

Angulation between the palatal plane and mandibular plane (NL-ML) was more with the RTBLP-RME (1.31) than with the FM-RME (0.77). Though the mandibular plane remained unchanged in the RTBLP-RME group, the divergence in the base plane could be a result of mild anteriorly upward tip of the palatal plane.

Ar-Go-Me showed mean change in RTBLP-RME (0.62), FM-RME (− 0.77), and control group (1.54). Opening in gonial angle was more with RTBLP-RME appliance than that with FM-RME appliance.

Changes in lower anterior face height (ANS-Me) were significant in overall comparison with mean change of RTBLP-RME group (4.08), FM-RME group (3.46), and control group (1.54).

Study by Baccetti et al. [18] recorded no significant change in the early treated group but a significant increase in lower anterior face height in the late treated face mask group.

### Measurements for assessment of condyle inclination

For angle Cond-AX-SBL, Cond-Ax-ML and Ar-Go-Vert T overall change was significant. Baccetti et al. [18] study with face mask .similar changes.

For condylar inclination, the findings are homogenous within both the treated groups showing posterior positioning of the condyle in the RTBLP-RME group and forward positioning of condyle with FM-RME group.

In RTBLP-RME bite was registered in the most retruded position which may be the cause of posterior position of condyle in glenoid fossa.

### Dentoalveolar measurements

Inclination of upper incisors (UI-SN) showed proclination in the RTBLP-RME group (3.08°), FM-RME group (5.46°), and control group (2.38°). A study by Masucci et al. [21] showed mean change of (2.4°) with face mask therapy.

Thus, with FM-RME, there was an increase dental compensation by upper incisor proclination along with alveolar bone remodeling. In the RTBLP-RME-treated group, the effect was more skeletal on the maxilla with significant maxillary advancement and minimal incisor proclination.

The findings of Seehra et al. [16] differ from the present study as maxillary incisor proclination was more in their study with reverse twin block compared to face mask. Kidner et al. [6] also found increase upper incisor proclination with reverse twin block.

Angle IMPA showed retroclination of lower incisors with FM-RME (− 0.62°) as compared to RTBLP-RME (0.23°).

Increased maxillary incisors proclination with decrease in mandibular incisor inclination with RTB therapy was reported by Kidner et al. [6] and Seehra et al. [16], suggesting primarily a dental effect with RTB in their study. With other removable functional appliance like removable mandibular retractor (RMR), similar findings have been reported [8–10].

In the present study, the overall evaluation period was short with no follow-up data. In fact, this is a limitation that should be further evaluated with larger sample size. Similarly, although in the present study we did not find any drop outs, the effect of compliance of patients in using these appliances is an area of future research.

Another limitation of the study was regarding the large age range. But because of some limitations (single center with low prevalence and time for study), we tried to take the average age range between early and late mixed dentition. This could have affected the clinical outcome of the appliances used and thereby the conclusion of the study.

## Conclusion

Based on clinical and cephalometric findings of two treatment groups when compared to control group, the following conclusions can be drawn:

1. From a clinical point of view, both modified RTB (RTBLP-RME) and FM-RME showed favorable results in 9 months but RTB (RTBLP-RME) had a greater impact on maxillary advancement and more hold on the posterior positioning of the mandible compared to FM-RME.
2. With RTBLP-RME, minimal dental compensation was observed compared with FM-RME.
3. For all vertical measurements, RTBLP-RME showed an increase compared to FM-RME which could be due to posterior open bite.

## Abbreviations

FM-RME: Face mask with rapid maxillary expansion appliance
RMR: Removable mandibular retractor
RTB: Reverse twin block
RTBLP-RME: Reverse twin block with lip pads and rapid maxillary expansion

## References

[1] Kharbanda OP. Orthodontics diagnosis and management of malocclusion and dentofacial deformities. 2nd ed. India: Elsevier; 2013. p. 547.

[2] Gu Y, Rabie AB, Hägg U (2000). Treatment effects of simple fixed appliance and reverse headgear in correction of anterior crossbites. Am J Orthod Dentofac Orthop.

[3] Seehra J, Fleming PS, DiBiase AT (2009). Orthodontic treatment of localized gingival recession associated with traumatic anterior crossbite. Aust Orthod J.

[4] Frankel R (1970). Maxillary retrusion in class III and treatment with the function corrector III. Trans Eur Orthod Soc.

[5] Loh MK, Kerr WJ (1985). The function regulator III: effects and indications for use. Br J Orthod.

[6] Kidner G, DiBiase A, DiBiase D (2003). Class III twin blocks: a case series. J Orthod.

[7] Seehra J, Fleming PS, Dibiase A (2010). Reverse twin block application for early dental class III correction. J Clin Orthod.

[8] Tollaro I, Baccetti T, Franchi L (1996). Craniofacila changes induced by early functional treatment of class III malocclusion. Am J Orthod Dentofac Orthop.

[9] Saleh M, Hajeer MY, Al-Jundi A (2013). Short-term soft- and hard-tissue changes following class III treatment using a removable mandibular retractor: a randomized controlled trial. Orthod Craniofac Res.

[10] Majanni AM, Hajeer MY (2016). The removable mandibular retractor vs the bone-anchored intermaxillary traction in the correction of skeletal class III malocclusion in children: a randomized controlled trial. J Contemp Dent Pract.

[11] Thilander B (1963). Treatment of angle class III malocclusion with chin cap. Trans Eur Orthod Soc..

[12] Sugawara J, Asano T, Endo N, Mitani H (1990). Long-term effects of chincap therapy on skeletal profile in mandibular prognathism. Am J Orthod Dentofac Orthop.

[13] Nartallo-Turley PE, Turley PK (1998). Cephalometric effects of combined palatal expansion and facemask therapy on class III malocclusion. Angle Orthod..

[14] Ngan P, Yiu C, Hu A, Hagg U, Wei SH, Gunel E (1998). Cephalometric and occlusal changes following maxillary expansion and protraction. Eur J Orthod.

[15] Cevidanes L, Baccetti T, Franchi L, McNamara JA, De Clerck H (2010). Comparison of two protocols for maxillary protraction: bone anchors versus face mask with rapid maxillary expansion. Angle Orthod.

[16] Seehra J, Fleming PS, Mandall N, Dibiase AT (2012). A comparison of two different techniques for early correction of class III malocclusion. Angle Orthod..

[17] Fareen N, Alam MK, Khamis MF, Mokhtar N (2017). Treatment effects of reverse twin-block and reverse pull face mask on craniofacial morphology in early and late mixed dentition children. Orthod Craniofac Res..

[18] Baccetti T, Franchi L, McNamara JA (2000). Treatment and posttreatment craniofacial changes after rapid maxillary expansion and facemask therapy. Am J Orthod Dentofac Orthop.

[19] Turely PK (2002). Managing the developing class III malocclusion with palatal expansion and face mask therapy. Am J Orthod Dentofac Orthop.

[20] Tortop T, Keykubat A, Yuksel S (2007). Facemask therapy with and without expansion. Am J Orthod Dentofac Orthop.

[21] Masucci C, Franchi L, Defraia E, Mucedero M, Cozza P, Baccetti T (2011). Stability of rapid maxillary expansion and facemask therapy: a long term controlled study. Am J Orthod Dentofac Orthop.

